# Recurrent Upper Extremity Thrombosis Associated with Overactivity: A Case of Delayed Diagnosis of Paget-Schroetter Syndrome

**DOI:** 10.1155/2017/8764903

**Published:** 2017-07-10

**Authors:** Himani Sharma, Abhinav Tiwari

**Affiliations:** Department of Internal Medicine, University of Toledo Medical Center, Toledo, OH, USA

## Abstract

Paget-Schroetter syndrome is thrombosis of the axillary-subclavian vein that is associated with strenuous and repetitive activity of the upper extremities. Overuse of the arm coupled with external compression results in microtrauma in the intima of the subclavian vein, resulting in the activation of the coagulation cascade. Diagnosis is usually made by Doppler ultrasound and the treatment involves thrombolysis, while routine surgical decompression of the thoracic outlet is controversial. In this report, we present a case of a patient who presented with a second episode of spontaneous right upper extremity deep venous thrombosis. The first episode was inadequately treated with oral anticoagulation alone. During the second episode, Paget-Schroetter syndrome was diagnosed, after careful review of his occupational history. He subsequently underwent angioplasty and decompression of thoracic outlet with no recurrence of thrombosis in a 12-month follow-up period.

## 1. Introduction

Paget-Schroetter syndrome (PSS) or “effort” thrombosis of the axillary-subclavian vein is an uncommon cause of deep vein thrombosis (DVT) seen in physically active and otherwise healthy individuals. It was first described by Paget in 1875 and Von Schroetter in 1884 and was named the “Paget-Schroetter syndrome” by Hughes in 1949 [1]. PSS accounts for 30–40% of spontaneous axillary-subclavian vein thrombosis (ASVT) and for 10–20% of all upper extremity deep venous thrombosis (UEDVT) [2]. Despite being a known cause of UEDVT, this entity is usually undiagnosed or misdiagnosed, mainly due to lack of awareness of the syndrome. Pathogenesis involves microtrauma to the intima of vasculature leading to intraluminal clot formation [3]. Diagnosis is usually made by Doppler ultrasound, computed tomography, and magnetic resonance venography. Oral anticoagulation alone is insufficient and catheter-directed thrombolysis (CDT) is usually performed [4]. Doing routine thoracic outlet decompression surgery following pharmacomechanical intervention is a topic of debate. In this report, we describe a case of a 38-year-old male patient who presented with second episode of right UEDVT in one year. Due to rigorous use of his right arm, PSS was suspected and he received pharmacochemical thrombolysis followed by first rib resection (FRR).

## 2. Case Report 

**Figure 1:**
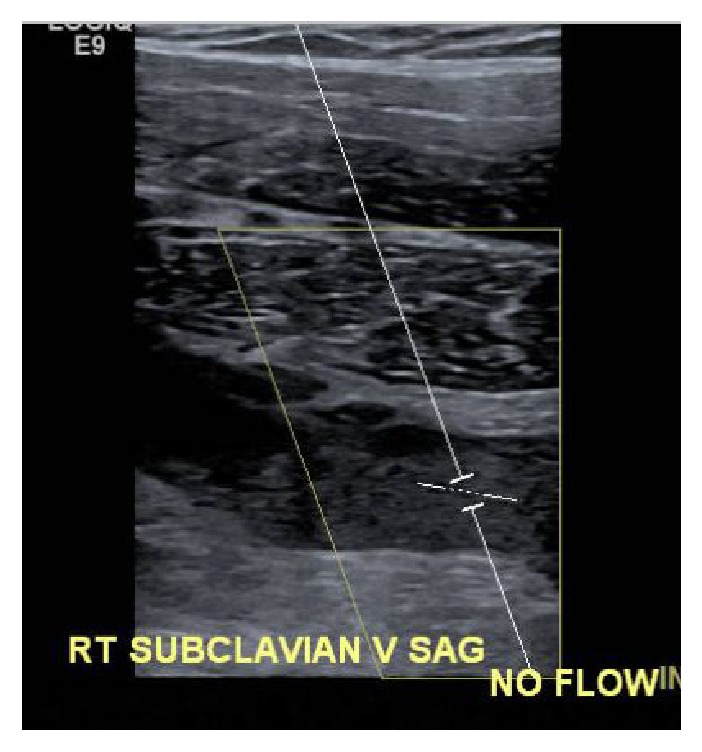
Doppler ultrasound showing absence of blood flow in the subclavian vein.

**Figure 2:**
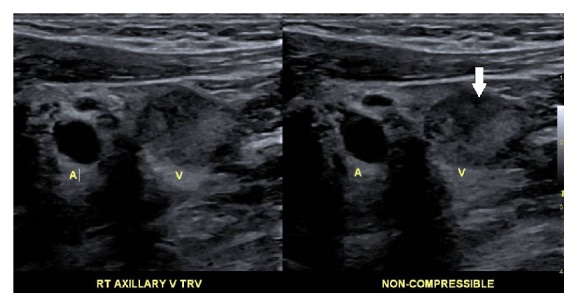
Doppler ultrasound showing noncompressibility of the axillary vein (arrow) due to thrombus.

**Figure 3:**
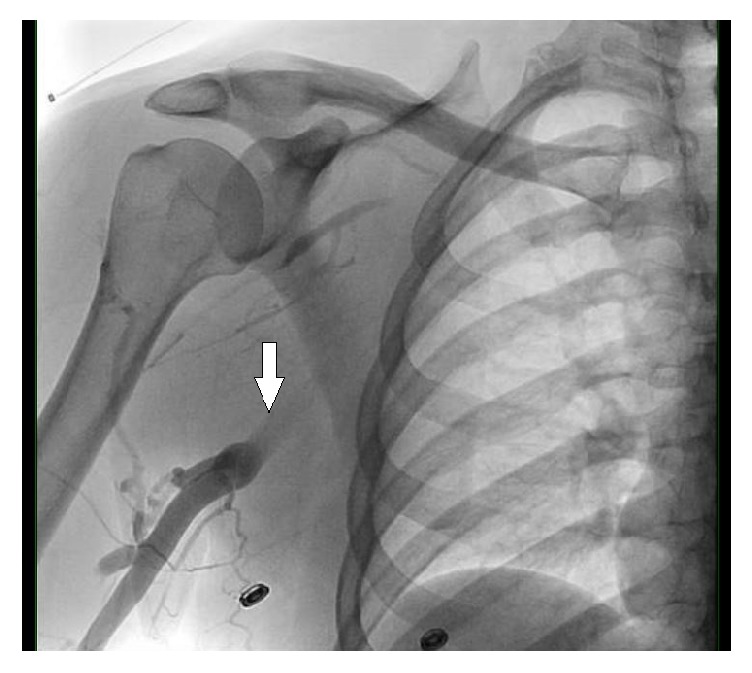
Angiogram showing abrupt cut-off in the contrast flow in the axillary vein (arrow) indicating obstruction.

**Figure 4:**
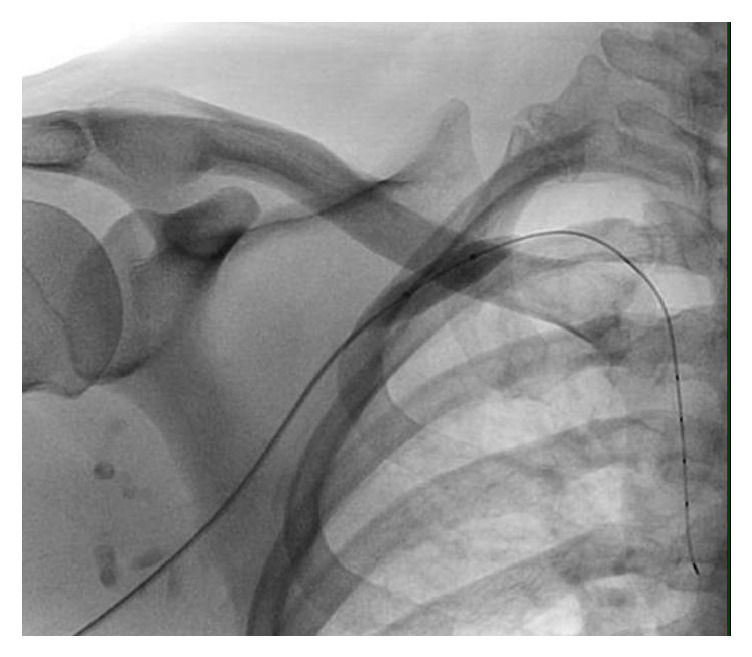
Fluoroscopic image showing balloon angioplasty being performed.

**Figure 5:**
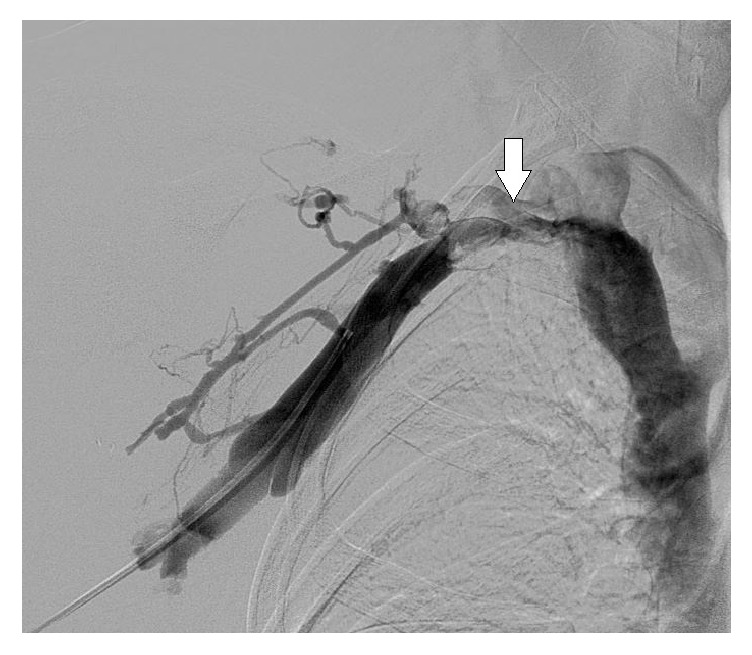
Peripheral angiogram showing residual stenosis at the level of clavicle (arrow) after performing thrombectomy and angioplasty.

A 38-year-old male patient was admitted with a two-week history of painful swelling of the right upper extremity. He denied any history of fever, rash, joint pain, or insect bite. He gave a history of a right UEDVT, diagnosed one year ago, treated with six months of oral rivaroxaban with no subsequent follow-up visits. On examination, he had a temperature of 37.5°C, a blood pressure of 128/78 mmHg, a pulse rate of 76 beats/minute, a respiratory rate of 18 breaths/minute, and an oxygen saturation of 98% on room air. On inspection, he had noticeable swelling and redness extending from his right wrist to the shoulder, and there was no superficially engorged vein on the arm or the chest. On palpation, he had mild tenderness of the affected area. All peripheral pulses were palpable and capillary refill time at right thumbnail was <2 sec. There was no palpable lymphadenopathy. All of his laboratory workup, including the complete blood count and coagulation profile, was unremarkable. A Doppler ultrasound of the right upper limb showed thrombosis of the right axillary, subclavian vein, and brachial veins (Figures 1 and 2), following which he was started on intravenous (IV) unfractionated heparin. The patient had no history of trauma and no personal history of malignancy or intravenous (IV) drug abuse. The etiology of the recurrent spontaneous DVT was initially unclear; however, on further questioning, the patient mentioned that he was a construction worker and his job involved a rigorous use of right arm with repetitive overhead labor. This additional information about his occupation led to the realization that PSS or effort thrombosis could be an etiology of ASVT in the context of arm activity. Urgent cardiology consult was obtained, and it was decided that the patient should undergo pharmacomechanical thrombolysis. Angiography revealed an abrupt cut-off in the contrast flow in the axillary vein (Figure 3). The AngioJet device was used for an initial run of thrombectomy followed by power pulse spray of tissue plasminogen activator (tPA) throughout the length of the thrombotic segment. Subsequently, thrombectomy was performed in multiple runs along the entire length of the thrombosed vein and repeat images showed a markedly improved contrast flow through the vein. However, there was some residual stenosis in the subclavian vein at the level of the first rib. Balloon venoplasty was then performed, using a 10 × 20 mm and subsequently using a 10 × 40 mm Charger balloon (Figure 4). Repeat venography revealed the presence of mild residual stenosis at the level of the first rib (Figure 5). Due to this residual stenosis, it was decided to surgically resect the first rib. During the surgical procedure, the subclavian vein was found to be thickened and had few collaterals, some of which were taken down. Intraoperatively, it was also noticed that the space between the first rib and the clavicle was extremely narrow and hypertrophied subclavius muscle was compressing the vein. Careful dissection of the subclavius muscle was performed following which the scalenus anterior and scalenus medius muscles were identified and removed in piecemeal. Lastly, the middle part of the first rib was transected. The patient tolerated the procedure well and was discharged on the second postoperative day on rivaroxaban for 6 months. The patient, at 6- and 12-month follow-up had no recurrence of symptoms while he continued his job as a construction worker.

## 3. Discussion

Upper extremity DVT can have a primary etiology, which accounts for about 20% of cases and includes either acquired or congenital anatomical anomalies. The remainder, 80% cases, are caused by secondary factors including the use of central venous or dialysis catheter, pacemaker insertion, and parenteral nutrition. Other secondary causes are the use of oral contraceptive pills, hypercoagulable state, and surgery on the upper arm [3]. Upper extremity DVT caused by secondary factors is usually treated with removal of the offending agent combined with anticoagulation use. Primary effort thrombosis or PSS is a rare entity with incidence between 1 and 2 per 100,000 population per year [3] and accounts for 1–4% of all venous thrombosis [4, 5]. About 60% to 80% of patients report repetitive and rigorous upper extremity activity, such as pitching, swimming, weight lifting, or even manual labor, at the onset of symptoms [6–8]. Therefore, it is more commonly seen in the dominant arm [9]. PSS is a sequela of thoracic outlet syndrome (TOS), as it involves compression of the subclavian vein, as it courses over the first rib, posterior to the clavicle in the anterior-most part of the thoracic outlet. Numerous factors can lead to its extrinsic compression, including anomalous subclavius or scalenus anterior, a long transverse process of the cervical spine, cervical rib, abnormal insertion of the first rib, congenital fibromuscular bands, or narrowing of the costoclavicular space from the depression of the shoulder [3, 10]. Subclavian vein is also subjected to intrinsic trauma due to repetitive shoulder-arm motion causing microscopic intimal tears in the vessel wall. It is postulated that chronic compression and microintimal trauma cause inflammation, which eventually leads to intimal hyperplasia and fibrosis of connective tissue surrounding the vein [11]. Fibrosis and scarring of tissue surrounding the vein decrease the mobility, thus making it susceptible to injury. Patients with effort thrombosis have an acute or subacute presentation and typically presents with a painful swollen and erythematous arm. On palpation, the arm is cold and tender with palpable venous cords [9, 12]. A duplex ultrasound scan is diagnostic with 56% to 100% sensitivity and 94% to 100% specificity for detecting DVT [8]. Once the diagnosis of PSS is established, a venography is recommended for both confirmation and therapeutics. A clot is usually visualized in the subclavian vein at the costoclavicular area or sometimes even more distally. The presence of collaterals also supports the diagnosis of PSS [3]. It is recommended to initiate treatment with parenteral anticoagulation when the diagnosis of PSS is confirmed [13]. Thrombolytic therapy is of most benefit to patients who have moderate-to-severe symptoms related to sudden axillosubclavian thrombosis. Catheter-directed pharmacological thrombolysis restores vein patency in 64 to 84% cases, with improved patency rates associated with earlier initiation of therapy [14]. Despite limited data on utility, mechanical thrombolysis (EKOS catheter, AngioJet) is often used in combination with pharmacologic thrombolysis [15]. Occasionally, percutaneous transluminal angioplasty (PTA) is performed to keep the vein open following thrombolysis if thoracic outlet decompression is contemplated [3]. There is conflicting data regarding routine resection of the first rib at the time of diagnosis of PSS. Some studies have shown that both nonsurgical and surgical groups show decent outcomes if the first rib is left alone [16–18]. However, many other observational studies suggest that there is a lower rate of recurrent thrombosis with reduced long-term morbidity with surgical decompression of the thoracic outlet, as compared to a more conservative approach [3, 19]. A meta-analysis showed that the symptom relief (95%) and vein patency rate (98%) were significantly more in the surgical group as compared to the group in which the rib is not removed (54% and 48%, resp.) [20]. In a retrospective study involving 22 patients with PSS, it was shown that pharmacomechanical thrombolysis followed by FRR had successful vein patency rate of 86% and all except 1 of the 22 patients were asymptomatic. The mean follow-up time was 25 ± 17 months [21]. In our case, the inciting events were repetitive endovascular trauma and external compression, which were not addressed during the first episode of DVT with anticoagulation alone; hence he presented with a second episode. In conclusion, occupational or recreational history plays a pivotal role in diagnosing PSS and failure to perform thrombectomy and surgical decompression may result in recurrent episodes of upper extremity DVT in such cases.
